# Minimally invasive versus open surgery for women with stage 1A1 and stage 1A2 cervical cancer: A retrospective database cohort study

**DOI:** 10.1016/j.amsu.2022.103507

**Published:** 2022-04-07

**Authors:** Judy Hayek, Mia Mowzoon, Saleshi Demissie, Albert Palileo, Eli Serur, Gary L. Goldberg, Ioannis Alagkiozidis

**Affiliations:** a Department of Obstetrics and Gynecology, Zucker School of Medicine at Hofstra/Northwell at Staten Island University Hospital, USA; b Department of Biostatistics, Feinstein Institutes for Medical Research, Staten Island University Hospital, Northwell Health, USA; d Department of Gynecologic Oncology, Zucker School of Medicine at Hofstra/Northwell at Staten Island University Hospital, USA; e Department of Obstetrics & Gynecology and Gynecologic Oncology, Zucker School of Medicine at Hofstra/Northwell at Long Island Jewish Medical Center and Feinstein Institutes for Medical Research, USA; f Department of Gynecologic Oncology, Maimonides Medical Center, USA

**Keywords:** Surgical outcomes, Minimally invasive hysterectomy, Cervical cancer stage IA, LACC trial, Overall survival, Length of stay, Readmission

## Abstract

**Background:**

Recent studies comparing minimally invasive versus open radical hysterectomy in patients with early-stage cervical cancer have reported a worse overall survival with minimally invasive surgery (MIS). However, in the patients with microscopic disease, there was no survival difference and the optimal surgical approach for microscopic cervical cancer remains unclear.

**Methods:**

Using the National Cancer Database, we identified a cohort of women who underwent hysterectomy as the primary treatment for stage IA1/IA2 cervical cancer between January 2010 and December 2016. Using multivariable logistic regression, our primary outcome was to compare overall survival between the open and MIS groups. The data was stratified for simple and radical hysterectomies. Secondary endpoint was comparison of readmission rates and length of stay (LOS).

**Results:**

We identified 6230 patients with stage IA1 and IA2 cervical cancer that underwent hysterectomy as primary treatment. 4054 of these women (65%) underwent MIS. There was no difference in age, lympho-vascular invasion, number of lymph nodes retrieved and histology between the two groups. In the overall cohort, there was no difference in survival between the open and the MIS group (Hazard ratio for the open group 1.23; CI 0.92–1.63). Post-operative radiation therapy was more common in the open group (5.24% vs 4.09%, p value < 0.02). The mean LOS (1.35 days vs 3.08 days) was shorter in MIS group (*p* value < 0.0001). No difference was found in the readmission rates (60% for the MIS group vs 55% for the open group; *p* value 0.14).

**Conclusions:**

Our data suggest that MIS is associated with similar overall survival and shorter length of hospital stay compared to the open hysterectomy in women with stage IA cervical cancer. Based on this large data set, MIS appears to be a safe and effective surgical approach for women with stage IA1/IA2 cervical cancer.

## Highlights

• Our study is the largest analysis to date on the use of MIS in stage-IA cervical cancer.
• There was no difference in overall survival between MIS and open surgery for stage IA cervical cancer.
• MIS was associated with a shorter length of stay. No difference was found in readmission rates.
• The two groups were similar for lympho-vascular invasion, number of lymph nodes retrieved, histology, and age.

## 1 Introduction

Women with early-stage cervical cancer are often treated with surgery. For patients with stage IA2-IIA1 who do not desire to preserve fertility, a radical hysterectomy with pelvic lymphadenectomy is the recommended surgical treatment [1,2]. For patients with stage IA1 with no lympho-vascular space invasion (LVSI), the risk of lymph node or parametrial involvement is minimal, and a simple, extra-fascial hysterectomy is recommended [1]. Based on the National Comprehensive Cancer Network [1] guidelines, the standard recommended surgical approach for a radical hysterectomy is an open abdominal approach [1]. This recommendation is based on the first randomized trial reported by Ramirez et al., in 2018 (LACC trial), showing lower disease-free and overall survival for patients undergoing minimally invasive radical hysterectomy for early-stage cervical cancer [3]. These findings were confirmed by epidemiologic studies demonstrating that minimally invasive radical hysterectomy was associated with shorter overall survival than open surgery [4].

The results of these studies have shaped the current standard of care but could not be generalized for women with microscopic disease. Stage IA accounted only for 51 out of 631 patients in the LACC trial and 10–13% of cases in the epidemiologic studies [3,4]. Therefore, these studies were not powered to evaluate survival outcomes in patients with microscopic disease. Currently, there is no published evidence that a minimally invasive surgical approach confers worse oncologic outcomes in patients with stage IA cervical cancer. Adequately powered studies specifically looking at this group of patients are needed. At the same time, retrospective studies have shown that minimally invasive surgery is associated with improved surgical outcomes such as decreased length of hospital stay and intra-operative blood loss [[5], [6], [7], [8], [9]].

We designed a retrospective study to evaluate the survival and surgical outcomes using a large cohort of patients from the National Cancer Database [10]. The primary objective was to compare the overall survival in patients with stage IA1 and IA2 disease who underwent minimally invasive to those who underwent open hysterectomy with further stratification for women who underwent a radical hysterectomy. Our secondary objective was to compare length of stay and hospital readmission rates.

## 2 Methods and materials

Data was obtained from the National Cancer Database, which includes data on patients who received some element of their cancer care (treatment or diagnosis) at a cancer program that is accredited by the Commission on Cancer- Accredited Centers [11]. Data covers more than 70% of newly diagnosed cases collected in approximately 1500 facilities. Institutional Review Board approval was obtained. (Protocol IRB #20–0556). Our data has been reported in line of the STROCSS criteria [12]. Our study was registered at the Chinese Clinical Trail Registry (ChiCTR2100050877).

We retrospectively identified a cohort of women who underwent simple or radical hysterectomy as the primary treatment for pathologically confirmed stages IA1/IA2 cervical cancer between January 2010 and December 2016. We excluded all patients who received radiation or underwent trachelectomy or cone excision as their primary treatment, those whose primary treatment was unknown, and those for whom there was a lack of pathological confirmation of cervical cancer. The documented surgical approach included open or minimally invasive (laparoscopic or robotic assisted) surgery. Our analysis was based on the intention-to-treat model in which we included any cases initiated as minimally invasive surgery even if they were converted to open surgery. Median length of follow up was 30 days for the length of stay and readmission variables. We compared the two groups in terms of age, race, co-morbidities, lympho-vascular invasion, number of lymph nodes removed during surgery and post-operative radiation therapy. Comorbid conditions were analyzed using the Charlson/Deyo Score provided by the National Cancer Database. The Charlson/Deyo value is a weighted score derived from the sum of the scores for each of the comorbid conditions listed in the Charlson Comorbidity Score; A score of 0 indicates “no comorbid conditions recorded”. Other conditions are listed in the appendix that reflect what scores they are given. Histology in the NCDB PUF dictionary was reported as ICD-O-3 codes reported by SEER registries.

The primary outcome was to compare the overall survival between patients undergoing minimally invasive (MIS) versus open surgical management. Secondary endpoints included the length of hospital stay and hospital readmission within 30 days of discharge. We further stratified the data for stages IA1 and IA2, separately. Similarly, we studied the data looking at simple and radical hysterectomy as separate cohorts. We also analyzed the survival outcomes for patients with stage IA2 who underwent MIS radical versus simple hysterectomy.

*Statistical Analysis:* Categorical data were summarized by the number and percentage of patients falling within each category. Continuous variables were summarized by descriptive statistics including mean and standard deviation or median and interquartile range. Bivariate analyses were performed using the χ^2^-test, two sample *t*-test and Mann–Whitney *U* test, as appropriate. Kaplan-Meier analysis with log-rank test and Cox proportional hazards regression were used to analyze time to first event of interest which was time to death. Data on regional lymph nodes examined were analyzed using zero-inflated Poisson [4] model. All statistical tests were two-sided. P-values <0.05 was considered statistically significant. All statistical analyses were performed using SAS software (Statistical Analysis Systems Inc., Cary, NC, USA). Confidence intervals (CIs) were also two-sided, unless otherwise stated.

## 3 Results

Between January 2010 and December 2016, we identified 6230 patients with stage IA1 and IA2 cervical cancer that underwent hysterectomy as their primary treatment. 4054 of these women (65%) underwent minimally invasive surgery. 1931 women had a radical hysterectomy and of those, 1152 had a minimally invasive radical hysterectomy (Table 1).

**Table 1:** Patient characteristics.

	OPEN	MIS	N = 6230
*Simple*	1397	2902	4299
*Radical*	779	1152	1931
*Stage IA1*	1337	2662	4002
*Stage IA2*	477	805	1282
*IA unspecified*			

Women who underwent minimally invasive hysterectomy, were more likely to be white. The mean age for both groups was similar (45.5 versus 46.3). The number of regional lymph nodes removed for examination was also reported and was found to be similar between the MIS group and the open group (2.93 vs 2.89 with a p value < 0.0001). We also compared the percentage of cases with lympho-vascular invasion (LVSI) between the two groups and found no statistically significant difference. 8.71% of patients who underwent minimally invasive hysterectomy were found to have LVSI, compared with 9.28% in the open surgery group (p value 0.40) (Table 2). Post-operative radiation therapy was more common in the open group (5.24% vs 4.09%, p value < 0.02) (Table 5). There was no clinical difference in the cervical histologic type between the two groups (p value = 0.0015). In the entire cohort, 3915 of the patients had squamous cell carcinoma, and of those 63.76% were in the MIS group versus 61.09% in the open group. In addition, 1840 had cervical adenocarcinoma of which 1.63% underwent MIS versus 1.07% open surgery. Neuroendocrine tumors only represented 0.36% of reported histology. A total of 23 neuroendocrine tumors were included. 0.27% of these women had MIS and 0.55% had open surgery (Table 6).

**Table 2:** Patient characteristics.

	Open	MIS	P value
Mean age	46.3	45.5	0.04
LVSI	9.28%	8.71%	0.49
No. lymph nodes removed	2.87	2.89	<0.0001

LVSI: lymphovascular stromal invasion.

### 3.1 Survival analysis

**Fig. 1:**
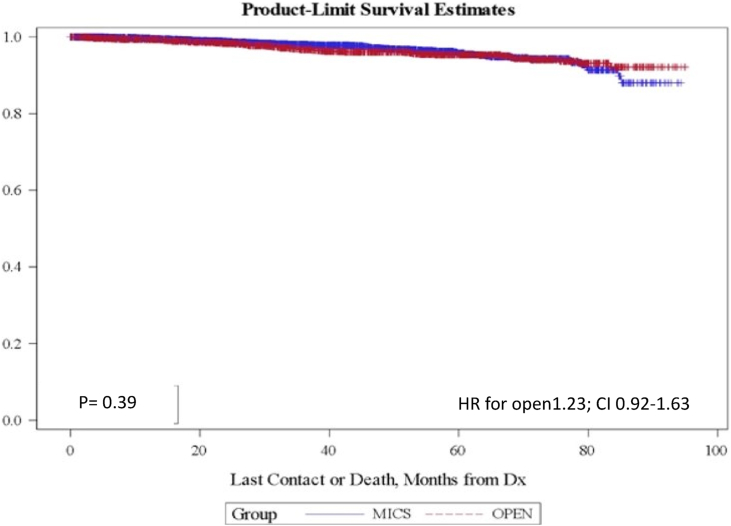
Overall survival.

There was no difference in the overall survival (OS) between the open and the MIS group (Hazard ratio for the open group 1.23; CI 0.92–1.63). 89 deaths (2.67%) occurred in the MIS group compared to 69 (3.59%) in the open group, but the difference was not statistically significant (p value 0.06). (Fig. 1). The mean length of stay was shorter in MIS group (1.35 days vs 3.08 days, *p* value < 0.0001). (Table 3). No difference was found in the readmission rates (60% for the MIS group vs 55% for the open group; *p* value 0.14) (Table 4).

**Table 3:** Hospital stay (P value < 0.0001).

	Mean	Standard deviation	Minimum	Maximum
MIS	1.35	3.22	0.00	96.0
Open	3.08	6.05	0.00	106.0

**Table 4:** Readmission rates (P value = 0.14).

Readmission	MIS	Open
Yes	112 (2.77%)	75(3.46%)
No	3927(97.23%)	2090(96.54%)

**Table 5:** Post-operative radiation (p value = 0.04).

Post-Operative radiation	MIS	OPEN
received	166 (4.05%)	114 (5.24%)
not received	3888 (95.91%)	2062 (94.76%)

**Table 6:** Histology (p value = 0.0015).

*Histology*	MIS	OPEN	TOTAL
*Squamous cell*	2585	1330	3915
*Adenocarcinoma*	1193	647	1840
*Adenosquamous*	66	32	98
*Neuroendocrine*	11	12	23
*Other*	199	156	355

For patients who underwent a radical hysterectomy, there was no difference in OS between the open radical and the minimally invasive radical cohorts (*p* value 0.31). As expected, the MIS group had a shorter length of stay. Mean of 1.76 days for the MIS group compared to 3.77 days in the open group (p value 0.001).

### 3.2 Cervical cancer stage IA1

There was no difference in OS for patients with stage IA1 disease (HR 1.1; CI 0.78–1.54). As expected, in women with stage IA1 cervical cancer, post-operative radiation was not as common as for patients with stage IA2. Those who underwent MIS compared to open surgery were less likely to receive radiation therapy post-operatively (2.21% versus 3.81% with p value 0.005). Mean length of stay for MIS group was 1.26 days compared to 2.78 days for the open group. (P value < 0.0001). There was no statistically significant difference in readmission rates between MIS and open groups (2.90% versus 3.83% with p value 0.13).

### 3.3 Cervical cancer stage IA2

For patients with stage IA2, there was a trend towards decreased OS in the open group that did not reach statistical significance (HR 1.77; CI 1.04–3.00). Similarly, length of stay for MIS group was shorter with mean of 1.62 days compared to 3.72 days for the open group. (P value < 0.0001). There was no difference in the readmission rates between the two groups (2.87% versus 4.01% with p value 0.33). Of note, there was no difference between the two groups in terms of receiving post-operative radiation. (9.19% versus 8.18% with p value 0.61).

### 3.4 Simple versus radical

4054 patients underwent MIS hysterectomy, of those 805 underwent a radical MIS hysterectomy. For this sub-cohort, we ran two analyses. First, we compared simple versus radical hysterectomy for all patients with stage IA2 disease. There was no difference in OS between the two groups with a hazard ratio for radical hysterectomy of 0.89: (CI 0.45–1.76). We then compared MIS simple hysterectomy versus MIS radical hysterectomy for the same stage group. Similarly, there was no difference in OS between the two groups. (HR 0.78; CI 0.29–2.01). Simultaneously, patients undergoing MIS radical hysterectomy were less likely to receive post-operative radiation (p = 0.0006).

## 4 Discussion

Our findings suggest that MIS was associated with a similar OS compared to open hysterectomy in patients with stage IA1/IA2 cervical cancer. MIS was associated with a shorter length of hospital stay. The results were the same for patients who underwent a radical hysterectomy. Prior studies have demonstrated worse oncologic outcomes with MIS in early-stage cervical cancer driven by patients with large volume disease [3,4]. However, we question whether these studies had power to draw such a conclusion.

In November 2018, Ramirez et al. published a phase 3, multi-center international randomized trial to study whether survival outcomes after minimally invasive radical hysterectomy were equivalent to those of open abdominal radical hysterectomy. The study results showed that the rate of disease-free survival at 4.5 years was 86% with MIS and 96.5% with open surgery. The was a hazard ratio of 3.74; 95% CI 1.63 to 8.58. The study also demonstrated a lower OS [3]. Concurrently, Melamed et al. conducted an epidemiological study using the National Cancer Database and SEER program database. In this study, 2461 patients with cervical cancer stage IA2 and IB1 were included; 1225 underwent MIS hysterectomy. Over a median follow-up of 45 months, the 4-year mortality was higher among women who underwent MIS with a hazard ratio of 1.65; 95% CI 1.22 to 2.22 (p = 0.002) [4]. These two pivotal studies represent a medical reversal; since their publication, the NCCN has adopted that the standard and recommended surgical approach for radical hysterectomy should be open abdominal [1]. Since their publication, several studies have reflected on the topic in hand, including, most recently, the SUCCOR study and the RACC trial. The SUCCOR study was a cohort observational study comparing disease-free survival in patients with IB1 cervical cancer undergoing MIS versus open radical hysterectomy [13], while the RACC trial is an ongoing trial with focus on comparing recurrence-free survival at 5 years between women who underwent robot-assisted laparoscopic surgery versus laparotomy for cervical cancer stage 1B1- IIA [14].

The above studies were not powered to evaluate patients with low-risk cervical cancer, including microscopic disease. In the LACC trial, only 10 (1.6%) patients had cervical cancer stage IA1, and 41 (6.5%) had stage IA2 [3]. Similarly, in Melamed et al., only 10–13% of the patients had stage IA disease. In this study, there was no survival difference in the subgroup analysis of patients with small-volume disease (<2 cm) [4]. Therefore, it is not possible to generalize the results of these studies to include microscopic disease. Conducting a randomized clinical trial to look at the oncological outcomes for stage IA1 and IA2 cervical cancer would require many years of national or international collaboration and may not be feasible. The next best alternative would be a high-powered retrospective analysis.

Recently, Wenzel et al. conducted a nationwide multicenter retrospective study analyzing data from the Netherlands Cancer Registry. They compared the laparoscopic and the abdominal approach to radical hysterectomy for women with cervical cancer stages IA2 with lympho-vascular invasion, IB1 and IIA1. A total of 1109 patient met the inclusion criteria. No difference was found between the two approaches in term of disease-free survival and OS [6].

Brandt et al. recently performed an analysis comparing radical MIS versus laparotomy for stage IA1, IA2 and IB1 at a nationally renowned large cancer center in New York [5]. The study included 196 patients who underwent surgery between 2007 and 2017. The results showed no difference in overall survival or 5-year disease free survival. All recurrences occurred in FIGO stage IB1 cases with residual tumor in the hysterectomy specimen and the differences were not statistically significant between the two groups. In our study, we offer similar data on overall survival in a setting of a much larger cohort.

In fact, to our knowledge, our study is the largest analysis to date on the use of MIS in stage IA cervical cancer. Moreover, we assessed several potential confounding factors between the study groups, such as the presence of LVSI, lymph nodes retrieved, patient comorbidities, and the use of post-operative radiation therapy. The treatment groups appear to be well balanced, except for post-operative radiation that is more common in the open group (5.24% versus 4.09%, p-value <0.02), for which further discussion below may suggest an explanation. According to our data, MIS provides similar OS with open surgery in patients with stage IA cervical cancer.

The reasons for the inferior oncologic outcomes in patients with macroscopic early-stage cervical cancer are not clear. Potential etiologic factors that have been implicated include the increased propensity for tumor spillage due to the uterine manipulator(3, 13) or an effect of CO2 on tumor cell spread and subsequent growth [15,16]. Some observational data supports these theories since intra-peritoneal spread or carcinomatosis were more common among patients with recurrence after MIS [17]. Interestingly, however, in the SUCCOR study, patients that underwent MIS without a uterine manipulator has similar relapse rates as patients who underwent open surgery [13].

In a study by Casarin et al., analyzing predictors of recurrence of cervical cancer stage 1A1-1B1 after laparoscopic hysterectomy, size of the tumor was seen to be an independent risk factor for recurrence [18]. Additionally, it has been shown that no recurrences were seen in patients with no residual disease on final pathology after radical hysterectomy [19]. This data supports the hypothesis that the metastasis pattern is associated with the tumor volume at the primary site, and it is reasonable to assume that microscopic tumors have less potential to spread through these routes and overcome the immune system leading to recurrent disease.

Another area of debate in the management of microscopic cervical cancer is the radicality of the surgery with the current recommendation for patients with stage IA1 with LVSI and stage IA2 is a radical hysterectomy(1). The surgical morbidity of a radical hysterectomy is significantly worse due to resection of the parametria; however, retrospective studies suggest that the risk for parametrial involvement in this group of patients is minimal and advocate for a simple hysterectomy [[20], [21], [22]]. There was no OS difference between patients with stage IA2 who had simple vs. radical hysterectomy in our study. As expected, use of post-operative radiation was more common in the group of patients who had a simple hysterectomy.

The finding of increased use of post-operative radiation after open surgery is subject to confounding effects. For example, radiation after open surgery would be recommended after disease upstaging from IA1 to pathologically confirmed IA2 and/or if there were no lymph node retrieval. This would be the case if a general gynecologist performed the surgeries. Without detailed information on the surgeons involved in the procedures and patient selection, this finding cannot be accurately interpreted.

We acknowledge several important limitations in our study. Although the NCDB captures women from many hospitals, these data may not be representative of the entire general population. Another important limitation of this study is the absence of information about disease recurrence, subsequent treatment at the time of recurrence, and cause of death in the National Cancer Database. The disease-specific mortality for patients with stage IA cervical cancer is very low, and the risk for recurrence would be the optimal measure of the efficacy of any intervention in this setting. Simultaneously, even if there is a difference in the risk of recurrence between the study groups, this has not translated to a difference in OS, and its magnitude should be minimal. Furthermore, we recognize that surgeon's experience has historically played a role in determining the radicality of the surgery as well as the approach, however, there is no information in the NCDB on the factors affecting the selection of patients between the open and MIS approach that could lead to potential selection bias. It may be suggested that their surgeons selected high-risk patients (based on pre-operative pathology or imaging) to undergo open surgery. However, our analysis found no difference in pathologic risk factors between the two groups. Finally, operative morbidity is a significant factor that affects the choice of the surgical approach, and our data lack this information.

In conclusion, our study includes a large cohort of patients with microscopic cervical cancer from a nationwide, multicenter database. Our data suggest that MIS is associated with similar OS compared to open hysterectomy with a shorter hospital length of stay. When the data was further stratified to simple and radical hysterectomy, the results were the same. Based on this large data set, MIS appears to be a safe and effective surgical approach for stage IA1/IA2 cervical cancer patients.

## CRediT authorship contribution statement

**Judy Hayek:** Investigation, Data curation, Validation, Writing – original draft, Visualization. **Mia Mowzoon:** Writing – review & editing. **Saleshi Demissie:** Formal analysis. **Albert Palileo:** Writing – review & editing. **Eli Serur:** Writing – review & editing. **Gary L. Goldberg:** Writing – review & editing. **Ioannis Alagkiozidis:** Writing – review & editing, Supervision, Project administration.

## Declaration of competing interest

There is no conflict of interest.

## References

[1] Abu-Rustum N.R., Yashar C.M., Bean S., Bradley K., Campos S.M., Chon H.S. (2020). NCCN guidelines insights: cervical cancer, version 1.2020. J. Natl. Compr. Cancer Netw..

[2] Cibula D., Pötter R., Planchamp F., Avall-Lundqvist E., Fischerova D., Haie Meder C. (2018). The European society of gynaecological Oncology/European Society for radiotherapy and Oncology/European Society of pathology guidelines for the management of patients with cervical cancer. Radiother. Oncol..

[3] Ramirez P.T., Frumovitz M., Pareja R., Lopez A., Vieira M., Ribeiro R. (2018). Minimally invasive versus abdominal radical hysterectomy for cervical cancer. N. Engl. J. Med..

[4] Melamed A., Margul D.J., Chen L., Keating N.L., Del Carmen M.G., Yang J. (2018). Survival after minimally invasive radical hysterectomy for early-stage cervical cancer. N. Engl. J. Med..

[5] Brandt B., Sioulas V., Basaran D., Kuhn T., LaVigne K., Gardner G.J. (2020). Minimally invasive surgery versus laparotomy for radical hysterectomy in the management of early-stage cervical cancer: survival outcomes. Gynecol. Oncol..

[6] Wenzel H.H.B., Smolders R.G.V., Beltman J.J., Lambrechts S., Trum H.W., Yigit R. (2020). Survival of patients with early-stage cervical cancer after abdominal or laparoscopic radical hysterectomy: a nationwide cohort study and literature review. Eur. J. Cancer.

[7] Diver E., Hinchcliff E., Gockley A., Melamed A., Contrino L., Feldman S. (2017). Minimally invasive radical hysterectomy for cervical cancer is associated with reduced morbidity and similar survival outcomes compared with laparotomy. J. Minim. Invasive Gynecol..

[8] Kim S.I., Cho J.H., Seol A., Kim Y.I., Lee M., Kim H.S. (2019). Comparison of survival outcomes between minimally invasive surgery and conventional open surgery for radical hysterectomy as primary treatment in patients with stage IB1-IIA2 cervical cancer. Gynecol. Oncol..

[9] Bogani G., Cromi A., Uccella S., Serati M., Casarin J., Pinelli C. (2014). Laparoscopic versus open abdominal management of cervical cancer: long-term results from a propensity-matched analysis. J. Minim. Invasive Gynecol..

[10] National Cancer Database [Internet] https://www.facs.org/quality-programs/cancer/ncdb.

[11] Surgeons ACo. CoC Quality of Care Measures [Available from: https://www.facs.org/quality-programs/cancer/coc/standards/2020.

[12] Mathew G., Agha R. (2021). STROCSS 2021: strengthening the reporting of cohort, cross-sectional and case-control studies in surgery. Int. J. Surg..

[13] Chiva L., Zanagnolo V., Querleu D., Martin-Calvo N., Arévalo-Serrano J., Căpîlna M.E. (2020). SUCCOR study: an international European cohort observational study comparing minimally invasive surgery versus open abdominal radical hysterectomy in patients with stage IB1 cervical cancer. Int. J. Gynecol. Cancer.

[14] Falconer H., Palsdottir K., Stalberg K., Dahm-Kähler P., Ottander U., Lundin E.S. (2019). Robot-assisted approach to cervical cancer (RACC): an international multi-center, open-label randomized controlled trial. Int. J. Gynecol. Cancer.

[15] Lin F., Pan L., Li L., Li D., Mo L. (2014). Effects of a simulated CO2 pneumoperitoneum environment on the proliferation, apoptosis, and metastasis of cervical cancer cells in vitro. Med. Sci. Monit..

[16] Volz J., Köster S., Spacek Z., Paweletz N. (1999). The influence of pneumoperitoneum used in laparoscopic surgery on an intraabdominal tumor growth. Cancer.

[17] Kong T.W., Chang S.J., Piao X., Paek J., Lee Y., Lee E.J. (2016). Patterns of recurrence and survival after abdominal versus laparoscopic/robotic radical hysterectomy in patients with early cervical cancer. J. Obstet. Gynaecol. Res..

[18] Casarin J., Buda A., Bogani G., Fanfani F., Papadia A., Ceccaroni M. (2020). Predictors of recurrence following laparoscopic radical hysterectomy for early-stage cervical cancer: a multi-institutional study. Gynecol. Oncol..

[19] Uppal S., Gehrig P.A., Peng K., Bixel K.L., Matsuo K., Vetter M.H. (2020). Recurrence rates in patients with cervical cancer treated with abdominal versus minimally invasive radical hysterectomy: a multi-institutional retrospective review study. J. Clin. Oncol..

[20] van Meurs H., Visser O., Buist M.R., Ten Kate F.J., van der Velden J. (2009). Frequency of pelvic lymph node metastases and parametrial involvement in stage IA2 cervical cancer: a population-based study and literature review. Int. J. Gynecol. Cancer.

[21] Sia T.Y., Chen L., Melamed A., Tergas A.I., Khoury-Collado F., Hou J.Y. (2019). Trends in use and effect on survival of simple hysterectomy for early-stage cervical cancer. Obstet. Gynecol..

[22] Stegeman M., Louwen M., van der Velden J., ten Kate F.J., den Bakker M.A., Burger C.W. (2007). The incidence of parametrial tumor involvement in select patients with early cervix cancer is too low to justify parametrectomy. Gynecol. Oncol..

